# Applications for the management of Attention Deficit Hyperactivity Disorder: a systematic review

**DOI:** 10.3389/fpubh.2025.1483923

**Published:** 2025-02-27

**Authors:** Maede Hosseinnia, Asiyeh Pirzadeh, Abouzar Nazari, Zahra Heidari

**Affiliations:** ^1^Department of Health Education and Promotion, School of Public Health, Isfahan University of Medical Sciences, Isfahan, Iran; ^2^Department of Health Education and Health Promotion, School of Public Health, Isfahan University of Medical Sciences, Isfahan, Iran; ^3^Department of Health Education and Promotion, School of Public Health, Tehran University of Medical Sciences, Tehran, Iran; ^4^Department of Biostatistics and Epidemiology, School of Health, Isfahan University of Medical Sciences, Isfahan, Iran

**Keywords:** children, attention deficit hyperactivity disorder, applications, APP, ADHD

## Abstract

**Background:**

Various interventions are available for managing Attention Deficit Hyperactivity Disorder (ADHD), including educational strategies and training programs. Recently, there has been a notable increase in the use of programs and apps as innovative tools to assist with ADHD management. This study aims to provide insights into the possibility of app-based therapies as a supplemental tool for ADHD care by analyzing the indicated advantages and supporting data.

**Methods:**

This systematic review was conducted until May 4, 2024, in several electronic databases, PubMed, MEDLINE, Scopus, Web of Science, and the Cochrane Central Register of Controlled Trials. In addition, we conducted a comprehensive search for relevant grey literature. The studies included trials, quasi-experimental studies, and observational studies on using apps to control ADHD. This includes studies that were published in English.

**Results:**

A review of 14 studies investigated the effectiveness of ADHD applications. Several applications monitored symptoms and enhanced cognitive function. Healthcare providers enhanced communication using the AKL-X01 app to monitor and record symptoms. The FOCUS ADHD app was well accepted but did not improve treatment compliance. The Sensory Diet App, known for its user-friendly interface, effectively alleviated symptoms associated with ADHD. BRUSH DJ enhanced dental hygiene and concentration on toothbrushing.

**Conclusion:**

Apps can potentially be adjunctive instruments for treating ADHD. Nevertheless, further study is required to validate their effectiveness over a long period and enhance their incorporation into complete treatment strategies for ADHD.

**Systematic review registration:**

identifier CRD42024523528 (PROSPERO).

## Introduction

Neurodevelopmental disease Attention Deficit Hyperactivity disease (ADHD) affects millions of children and people around the world (1). People with ADHD do a lot more than “not pay attention.” Millions of people around the world have this common neurological disease (2). ADHD makes it hard for people to focus, be hyperactive, and control their impulses, which can affect everything from schoolwork to relationships (3). The number of persons with ADHD is high. Studies show that around 10% of children and adolescents have ADHD, and the condition may persist into adulthood (4–6).

Approximately 7.2% of children in the world are diagnosed with ADHD, where the prevalence among boys is greater than in girls, at 10 and 5%, respectively (7). The prevalence of ADHD has emphasized that it is an important neurobehavioral disorder in Asian children and adolescents; however, the diagnosis and treatment method usually differs because of cultural and environmental factors. Depending on the diagnostic technique, ADHD is found to affect a significant fraction in the Iranian population. In one study, a frequency of 11.3% in Hamadan elementary school children was recorded; boys-19.4%, showing greater rates compared to girls.

Comprehending the significance of ADHD extends beyond just numerical data. Untreated ADHD may result in academic challenges, social seclusion, and diminished self-confidence (8). Nevertheless, if given a proper diagnosis and sufficient support, people with ADHD may thrive (9). A complete approach is necessary for an optimum treatment plan for ADHD, which often involves combining medication and non-pharmacological therapy (10). While medication is still a common treatment option, there has been a recent upsurge in the usage of programs or apps as creative tools to help manage ADHD (11).

These applications differ in their scope and are designed to target various demographic groups. Some help children with ADHD stay on track academically, while others teach parents how to manage their child’s behavior (12). Some apps assist people with ADHD stay organized and focused at work. The features provided by these applications are equally varied (13). Certain individuals may use timers and reminders to ensure users adhere to their schedules, whilst others may include gamified components to enhance the appeal of maintaining attention (14). Some applications include cognitive behavioral therapy (CBT) components to assist users in developing coping strategies for controlling their ADHD symptoms (15).

However, the question remains: Do these applications hold real promise for effectively managing ADHD, or are they just a passing trend that will eventually lose popularity? That is the exact issue our systematic study seeks to investigate. We’ll be delving further into the realm of ADHD applications, looking at the many alternatives available, the target audience for each, and the benefits each one provides. Above all, we’ll analyze the data to see if these applications deliver on their promises and may be a valuable addition to the ADHD treatment toolset. This study aims to provide insights into the possibility of app-based therapies as a supplemental tool for ADHD care by analyzing the indicated advantages and supporting data.

## Methods

The Preferred Reporting Items for Systematic Reviews and Meta-Analyses (PRISMA) standards were followed in this systematic review (16). It was also registered on PROSPERO (International Prospective Register of Systematic Reviews; PROSPERO Code No: CRD42024523528).

### Search strategy

A systematic search (until May 4, 2024) of several electronic databases, such as PubMed, MEDLINE, Scopus, Web of Science, and the Cochrane Central Register of Controlled Trials (CENTRAL), was conducted. In addition, we conducted a comprehensive search for relevant grey literature using resources such as OpenGrey and conference proceedings databases. The search approach included phrases about ADHD applications and keywords for “applications” and “Non-attention deficit hyperactivity disorder.” The search was refined and ensured a complete capture of relevant research by using Boolean operators (AND, OR). An example search method for PubMed is shown in the appendix. A tailored search method was built for each database used. In this study, the PICOS for the review will be specified as follows: population-P defined as adults and children diagnosed with ADHD; intervention-I reviewed in terms of using educational applications designed to deal with challenges associated with ADHD; comparison-C defined as people without ADHD control or reference group. The outcomes, or O, include improvements related to symptoms of inattention, hyperactivity, and impulsivity; academic achievement; social interactions; and overall quality of life. The intervention study design, described as S, includes trials or quasi-experimental studies that will be used to evaluate the effectiveness of the interventions in a systematic manner.

### Selection criteria

Studies must meet the following requirements to be eligible for inclusion in this evaluation. Original research publications (trials, quasi-experimental studies, and observational studies) on using apps to control ADHD were considered for inclusion. Case studies, reviews, conference lechers, posters, and comments are exempt. This evaluation relies heavily on established criteria, such as the DSM-5 and the ICD-10, to categorize patients with ADHD. We considered studies concentrating on specific age groups, such as children and adults. During the research, an application developed for the treatment of ADHD was evaluated. Studies should report on outcomes related to symptoms of attention-deficit/hyperactivity disorder (such as inattention, hyperactivity, and impulsivity), academic achievement, functions linked to social interaction, or quality of life. This includes studies that were published in English.

### Data extraction and management

Based on the set guidelines for what to include and what to leave out, two authors reviewed the titles, descriptions, and full texts of collected studies. Discussions or talks with a third reader settled any differences. A standardized data extraction form (in Excel format) was utilized to obtain pertinent information from the included studies. This contained information about the research design, participant characteristics, app features, and reported results. Two reviewers extracted data separately and resolve differences by consensus.

### Data synthesis

The studies were merged using a narrative synthesis. This paper thoroughly reviewed the applications’ features, targeted users, feasibility, and reported results. We also reviewed the benefits and downsides of the study methodology utilized and the general robustness of the evidence on the effectiveness of app-based therapy for ADHD.

## Results

### Search

**Figure 1:**
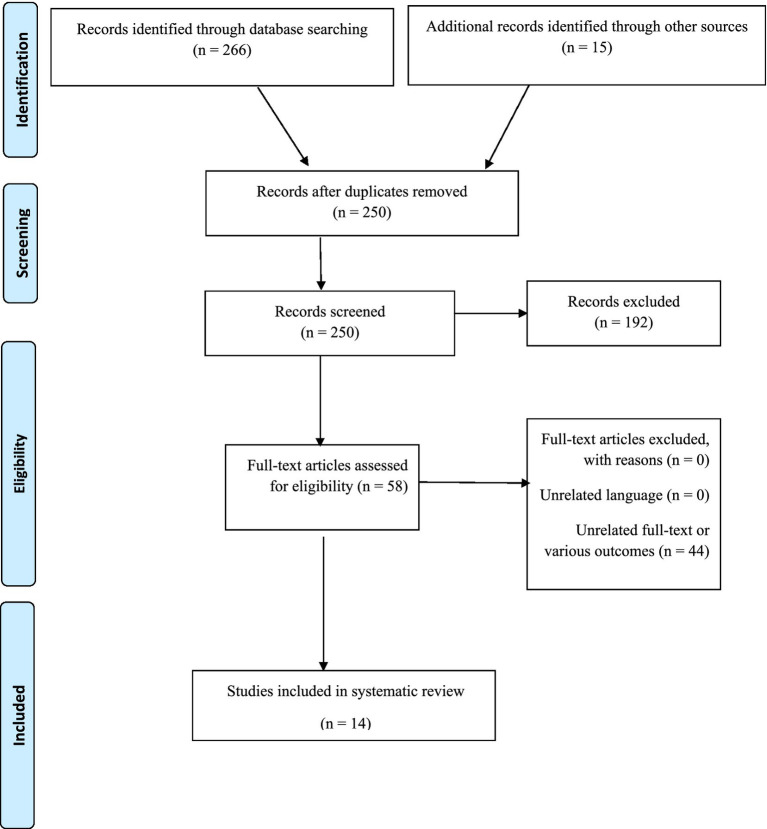
Flow diagram of the literature search and study selection.

Our initial search found 266 articles from databases and 15 from other sources potentially relevant studies. We then carefully reviewed these studies by removing duplicates and examining titles, abstracts, and even full texts. This process narrowed our selection down to just 14 articles that are most relevant to this review (see Figure 1 for details).

### Articles characteristics

Table 1 highlights 14 research from varied demographic situations that shed information on ADHD-focused apps’ efficacy. These research explored diverse participant demographics in the US, India, Spain, South Korea, Germany, Iran, Romania, and Israel. Most therapies targeted children and adolescents with ADHD, while some included adults and caregivers. Sample sizes ranged from 12 to 200 participants. Most study designs were trial-based or quasi-experimental, while some used mixed-methods to collect qualitative data. Most research participants were male, according to Delgado-Gómez. These treatments targeted symptom management, treatment adherence, behavioral monitoring, and caregiver and educator support. Reduced ADHD symptoms, improved inattention and hyperactivity, executive functioning, academic success, and medication adherence showed the potential advantages of these applications.

**Table 1:** Summary of Studies on ADHD Applications (*n* = 14).

Row	Year	Country	First author	Sample size	Design	Participants	App purpose	App name	Reported outcomes
1	2019	USA	J. Abraham (17)	125	Quasi-experimental	Caregivers of children/adolescents with ADHD (7–17 years old)	Track behaviors and symptoms	AKL-X01	Positive caregiver feedback, improved tracking, better communication with healthcare providers
2	2023	India	L. R. Carvalho (20)	73 (3 groups)	Trial	Adults with ADHD	Improve treatment adherence	FOCUS ADHD	Increased medication registration (with incentive), high adoption rate, no significant improvement in adherence or knowledge
3	2020	Spain	D. Delgado-Gómez (29)	32 (29 males)	Trial	Children with ADHD	Improve inattention severity	Running Raccoon	Correlation between game performance and inattention scores
4	2023	India	H. Gurnani (22)	54	Trial	Children with ADHD	Facilitate oral hygiene practices	BRUSH DJ	Improved oral hygiene and attention during brushing
5	2022	South Korea	S. Ha (24)	21 (12 ADHD, 9 ID)	Quasi-experimental	Children with ADHD and/or ID	Improve attention and cognition	DoBrain	Changes in neural activity, improved executive function, attention, and hyperactivity-impulsivity
6	2023	Iran	S. Khanahmadi (18)	24	Trial	Children with ADHD	Reduce ADHD symptoms	Sensory Diet App	High usability rating, improved symptoms in intervention group
7	2021	USA	S. H. Kollins (25)	206 (130 intervention)	Trial	Children with ADHD (8–14)	Improve inattention	AKL-T01	Improved impairment rating scale in both groups
8	2024	Korea	S. Y. Kwon (30)	74 (35 intervention)	Trial	Children with ADHD (8–15)	Improve symptoms, attention, and executive functions	OMNIFIT BRAIN	Improved general and auditory attention (mobile neurofeedback group)
9	2023	Romania	C. R. Pasarelu (31)	21	Mixed-methods	Parents of children with ADHD	Support behavioral parent training	ADHD Coping Card	Perceived usefulness of app, high user rating for friendliness
10	2016	USA	S. Schuck (23)	12	Quasi-experimental	Students with ADHD (ages 9–11)	Support classroom behavior management	iSelfControl	Promising for self and teacher evaluation
11	2023	Germany	B. Selaskowski (19)	34 (17 each group)	Trial	Adults with ADHD	Treat adult ADHD	Not Mentioned	Reduced ADHD symptoms (both app and chatbot)
12	2022	Germany	B. Selaskowski (21)	60 (30 each group)	Trial	Adults with ADHD	Psychoeducation	Not Mentioned	Reduced ADHD symptoms, improved inattention and impulsivity, higher homework compliance (app group)
13	2022	Iran	A. Sheikhtaheri (26)	12	Quasi-experimental	Parents of children with ADHD	Improve ADHD symptoms with sensory diet	Not Mentioned	High usability rating
14	2018	Israel	O. Weisman (32)	39 (27 boys)	Trial	Children with ADHD	Improve medication adherence	Not Mentioned	Increased medication adherence and improved ADHD symptoms

### Outcome

The The trials used several applications that targeted different facets of ADHD, including symptom monitoring, medication adherence, cognitive training, specific functioning, and behavior management (see Table 1 for details).

Symptom monitoring: The AKL-X01 app showed the potential to assist caregivers in tracking behaviors and symptoms of children and adolescents with ADHD. Because of this improvement in tracking, there was an improvement in contact with healthcare practitioners (17). The Sensory Diet App (18) showed promise in reducing ADHD symptoms in children, and participants found it to be user-friendly. Similarly, studies on apps for adults with ADHD (11, 12, 19) reported reduced ADHD symptoms in both app and comparison groups (chatbot and psychoeducation).

#### Medication adherence

The FOCUS ADHD app showed good adoption rates and increased medication registration (20), but it did not significantly improve overall treatment adherence or knowledge. There was no substantial improvement in any of these categories.

#### Cognitive training

The Sensory Diet App (18) has shown potential in mitigating ADHD.

symptoms in youngsters, and participants reported it to be easily navigable. Similarly, research conducted on applications designed for adults with ADHD (19, 21) found that both the app group and the comparator groups (chatbot and psychoeducation) had a decrease in ADHD symptoms.

#### Specific functioning

BRUSH DJ (22) was effective in improving oral hygiene and attention during brushing in children with ADHD.

#### Behavior management

While limited in scope, Schuck (23) found the iSelfControl app promising for self and teacher evaluation in supporting classroom behavior management for students with ADHD.

## Discussion

This systematic review examined the present state of apps created explicitly for managing ADHD. We have discovered 14 studies that examine the efficacy of these applications in different areas of ADHD, such as symptom monitoring, medication adherence, cognitive training, specific functioning, and behavior management. While the overall results look promising, further analysis of the findings reveals subtleties that could inform future research and app development.

The review shows several results that are encouraging. Applications such as AKL-X01 enable caregivers to monitor their patients’ actions and symptoms, improving communication with medical professionals (17). In the same way, BRUSH DJ showed efficacy in enhancing the dental hygiene and concentration of youngsters with ADHD when brushing (22). Investigation using DoBrain and Running Raccoon indicates that cognitive function may have advantages; DoBrain exhibits improvements in executive function, attention, hyperactivity-impulsivity, and alterations in brain activity (24).

Furthermore, several studies reported reductions in ADHD symptoms with various applications. The Sensory Diet App showed promise in this area, and participants found it user-friendly (25). Remarkably, research on applications for individuals with ADHD has shown that both the app and comparator groups (chatbot and psychoeducation) had fewer symptoms (19, 21). This implies that using an app might have some therapeutic benefits in addition to the particular features provided.

The fundamental symptoms of ADHD, such as inattention and impulsivity, did not demonstrate substantial improvement in specific trials. The FOCUS ADHD app did not substantially enhance overall treatment adherence or knowledge (20), although it had a high adoption rate and improved the number of people who registered for medication. The significance of these results depends on the fact that more study is required to optimize app functionality and target particular applications appropriately.

Applications like AKL-X01 and BRUSH DJ thus offered enormous advantages in their respective spheres of application—symptom tracking and improvement in dental hygiene (17, 22). These successes suggest that targeted app functionalities, when they meet specific needs—such as improvement in communication for caregivers or integration of routine behaviors like brushing—are capable of yielding substantial benefits (17, 22). On the other hand, an application like FOCUS ADHD, though highly adopted, was not successful in bringing large-scale improvements in either treatment adherence or knowledge (20). This may be due to the possible difference in design, target population, and even motivational frameworks used by the application.

User-friendly design allowed apps like the Sensory Diet App to be able to work (25). High usability scores are associated with better results at symptom management, likely facilitated by ease of access and appealing interfaces. However, adoption in the various studies was highly variable, raising questions about cultural, demographic, and technological factors that may affect the acceptance of such technology. For example, although FOCUS ADHD was very highly registered, the modest effect on adherence underlines the importance of combining usability with potent behavioral interventions (20).

According to the USA, Germany, India, and Iran, significant differences between countries with regard to app outcomes demonstrate strong effects of cultural and environmental factors on app outcomes (19, 21, 23, 26). This probably means that divergent health care infrastructures, different states of digital literacy, and different societal views on mental health may influence acceptance and therefore effectiveness (27, 28). For example, German psychoeducation apps or those integrating a chatbot show positive effects in that country from a cultural perception point of view with a focus on self-guided learning. Future studies need to be directed at exploring or investigating how localization of content and features can lead to better adoption and effectiveness.

### Limitations and future directions

Several limitations should be considered when interpreting these findings. First, the studies used different methodologies, and the sample sizes of some were relatively limited. Second, most of the studies concentrated their scope on narrow age groups, either children or adults, which may have prevented the generalization of their results across wider sections of age groups. In addition, the efficacy of many uses over the long term is not yet known. Any efficacy of these smartphone apps in the therapy of ADHD would be usefully complemented by further comprehensive and diverse study. Future studies should investigate long-term effects of using the app and the ability to embed such apps into wider approaches to treating ADHD at all ages.

Additionally, researchers should examine the possible pathways underlying the stated benefits. Can self-monitoring, gamification, or other methods improve application results? Understanding these methods may help developers build more focused and profitable apps.

## Conclusion

Finally, future research should consider the cost-effectiveness and accessibility of these apps. Ensuring the affordability and accessibility of applications is critical for people and families managing ADHD, notwithstanding their potential advantages.

Ultimately, apps can potentially be adjunctive instruments for treating ADHD. Various applications showcase beneficial impacts on specific facets of ADHD. Nevertheless, further study is required to validate their effectiveness over a long period and enhance their incorporation into complete treatment strategies for ADHD.

## Data Availability

The original contributions presented in the study are included in the article/supplementary material, further inquiries can be directed to the corresponding author.
